# Assessment of imprinting- and genetic variation-dependent monoallelic expression using reciprocal allele descendants between human family trios

**DOI:** 10.1038/s41598-017-07514-z

**Published:** 2017-08-01

**Authors:** Trees-Juen Chuang, Yu-Hsiang Tseng, Chia-Ying Chen, Yi-Da Wang

**Affiliations:** Genomics Research Center, Academia Sinica, Taipei, Taiwan

## Abstract

Genomic imprinting is an important epigenetic process that silences one of the parentally-inherited alleles of a gene and thereby exhibits allelic-specific expression (ASE). Detection of human imprinting events is hampered by the infeasibility of the reciprocal mating system in humans and the removal of ASE events arising from non-imprinting factors. Here, we describe a pipeline with the pattern of reciprocal allele descendants (RADs) through genotyping and transcriptome sequencing data across independent parent-offspring trios to discriminate between varied types of ASE (e.g., imprinting, genetic variation-dependent ASE, and random monoallelic expression (RME)). We show that the vast majority of ASE events are due to sequence-dependent genetic variant, which are evolutionarily conserved and may themselves play a *cis*-regulatory role. Particularly, 74% of non-RAD ASE events, even though they exhibit ASE biases toward the same parentally-inherited allele across different individuals, are derived from genetic variation but not imprinting. We further show that the RME effect may affect the effectiveness of the population-based method for detecting imprinting events and our pipeline can help to distinguish between these two ASE types. Taken together, this study provides a good indicator for categorization of different types of ASE, opening up this widespread and complex mechanism for comprehensive characterization.

## Introduction

Genomic imprinting is an epigenetic process through which genes are expressed in a parent-of-origin-specific manner. Accumulating evidence reveals its important role in varied diseases including cancer and numerous neurological and psychiatric disorders^1–3^. The gold standard for detection of mammalian imprinting is based on high-throughput transcriptome sequencing (RNA-seq) data from reciprocally crossed F1 hybrids of diverged inbred animal strains^4–7^. The maternal (or paternal) imprinting is determined as allele-specific expression (ASE) consistently biased toward the paternal (or maternal) allele in reciprocally crossed F1 hybrids. This reciprocal mating approach has been widely practiced in mice. However, such a mating system of engineered crosses cannot be applied to humans. In addition, ASE events may arise from other factors such as various types of random monoallelic expression (RME)^8, 9^ or *cis*-regulation of genetic variants^10–13^. Although numerous genome-scale methods based on RNA-seq data from multiple tissues or large family samples have been developed and identified scores of human imprinted gene loci^14–19^, the discrimination between different types of ASE is still challenging. Recently, the population-based approach^20, 21^, which identifies imprinted genes by examining the distribution of ASE between the reference and alternative alleles across individuals, has been successfully employed in detecting human imprinted genes. Such a population-based analysis is particularly powerful for eliminating varied confounding factors that result in ASE in specific individuals^21, 22^. Nevertheless, this approach is congenitally hampered by the paucity of both population-scale RNA-seq and genome-sequencing (or genotyping) data of a single individual.

Moreover, the genetic allelic effect such as expression quantitative trait loci (eQTLs) have been reported to be prevalent in mammals^23–26^, which can result in allelic difference in chromatin and thus allelic expression imbalance^27–29^. Such *cis*-regulated events often masquerade as imprinting because of ambiguous ASE effects of genetic variation in genic regions. Particularly, a previous study has showed that imprinting detection based on high-throughput transcriptome sequencing is subject to noises in the experimental approach and assay, leading to overestimation of imprinted genes^30, 31^. Therefore, there remains a need for a systematical method capable of effectively distinguishing imprinted events from other types of ASE events.

**Figure 1:**
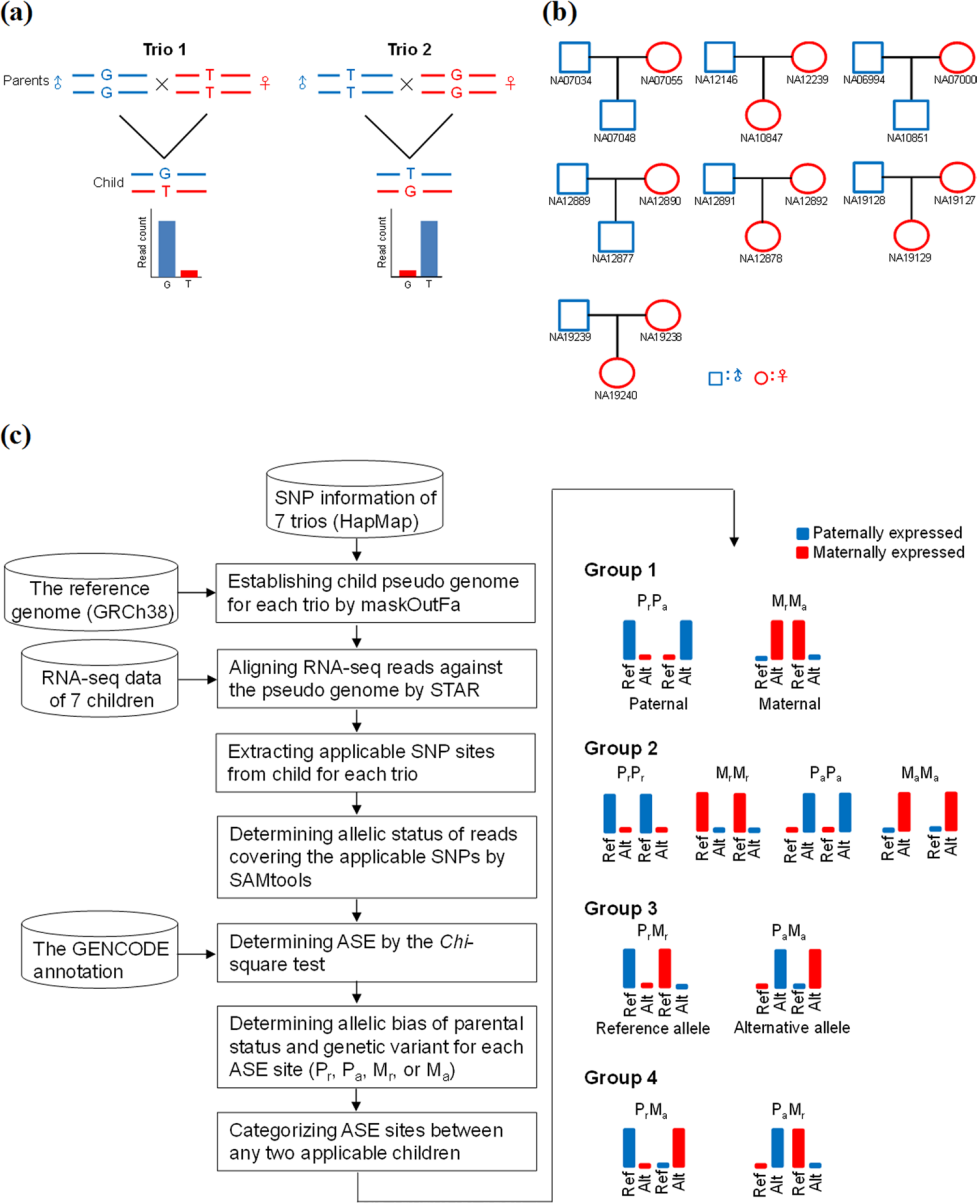
Identification of varied types of ASE patterns between unrelated family trios. (**a**) Schematic illustration of the RAD pattern. ASE consistently biased toward the paternal allele between two independent parent-offspring trios (i.e., Trios 1 and 2), without regard to genetic variation. (**b**) The seven family trios used in this study. (**c**) Flowchart of identification (left) of ASE events and categorization (right) of different groups of ASE patterns between any two children (see the text). P_r_, ASE biased toward the paternal and reference allele. P_a_, ASE biased toward the paternal and alternative allele. M_r_, ASE biased toward the maternal and reference allele. M_a_, ASE biased toward the maternal and alternative allele. Ref, the reference allele. Alt, the alternative allele.

Recently, the 1000 genomes project has provided the genotype data of numerous family trios (mother, father, and child) from human lymphoblastoid cell line (LCL) samples^32, 33^. The corresponding RNA-seq data of some LCL samples from the same individuals were also generated and publicly available^34–36^. Integration of these data enables us to extract the pattern of reciprocal allele descendants (RADs) between family trios and then identify parent-of-origin-dependent ASE sites by controlling for potential allelic expression and/or parental biases. Conceptually, the RAD-based approach is similar to reciprocal mating approach used in mice. An example of maternally imprinted/paternally expressed event is given in Fig. 1a, in which ASE consistently biases toward the paternal allele between two independent parent-offspring trios (i.e., Trios 1 and 2) without regard to genetic variant. We further distinguish between imprinting, genetic variation-dependent ASE, and RME events by comparing the combinations of ASE patterns between family trios. This study thus provides a good indicator for categorization of varied types of ASE events, opening up the important and complex mechanism of ASE for comprehensive characterization.

## Results

### Identification and categorization of ASE events

To identify RAD-based ASE events, we retrieved seven family trios (Fig. 1b), in which all members of each trio family were genotyped and the corresponding RNA-seq data of the child of each trio family should be available. The applicable SNPs were then extracted from the seven children (Table 1, see Materials and Methods). Sites of ASE (“ASE sites”) were then determined by using the *Chi*-square test on the mapped reads at the applicable SNPs (Fig. 1c; Materials and Methods). According to the allelic bias of parental status (maternal (designated “M”) or paternal (designated “P”) alleles) and genetic variant (reference (designated “r”) or alternative (designated “a”) alleles), each ASE site can be classified into one of the four types: P_r_, P_a_, M_r_, or M_a_. To minimize the effect of partial imprinting or heterogeneous imprinting across individuals^19, 21, 37^, we only considered the ASE sites that exhibited the ratio of the read count of the higher expressed allele to that of the other allele over two with a *Chi*-square test *P* value < 10^−3^ (Table 1). There are 10 possible combinations of ASE patterns between any two applicable children, which can be classified into four groups (Fig. 1c, right):

**Table 1:** The applicable SNP sites and identified ASE events for each trio.

Family trio			Genotyped Applicable			ASE sites	
Child (gender)	Father	Mother	Race	SNP	SNP^a^	*P* < 0.05^α^	*P* < 0.001^c^
NA07048 (M)	NA07034	NA07055	Caucasian	4,030,563	307,260	259	89
NA10847 (F)	NA12146	NA12239	Caucasian	4,030,563	656,869	1,053	301
NA10851 (M)	NA06994	NA07000	Caucasian	4,030,563	226,794	1,063	790
NA12877 (M)	NA12889	NA12890	Caucasian	4,030,563	340,619	853	145
NA12878 (F)	NA12891	NA12892	Caucasian	4,030,563	666,998	964	276
NA19129 (F)	NA19128	NA19127	Yoruba	3,984,147	716,574	843	238
NA19240 (F)	NA19239	NA19238	Yoruba	3,984,147	718,802	3,797	994

^a^The heterozygous SNPs that were informative for analysis in the child of a trio family (see Materials and Methods).

^b^The heterozygous SNPs exhibited imbalanced expression between two alleles (*P* value < 0.05 by the *Chi*-square test).

^c^The heterozygous SNPs exhibited imbalanced expression between two alleles, with the ratio of the read count of the higher expressed allele to that of the other allele over two and ASE score > 3 (*P* value < 0.001 by the *Chi*-square test).

Group 1: ASE consistently biased toward either the paternal (P_r_P_a_) or maternal (M_r_M_a_) allele with the RAD pattern but without regard to genetic variant

Group 2: ASE consistently biased toward the same allele without the RAD pattern (e.g., P_r_P_r_, M_r_M_r_, P_a_P_a_, or M_a_M_a_)

Group 3: ASE consistently biased toward either the reference (P_r_M_r_) or alternative (P_a_M_a_) alleles with the RAD pattern but without regard to parent of origin

Group 4: ASE biases occurred without regard to parent of origin or genetic variant (P_r_M_a_ or P_a_M_r_).

**Figure 2:**
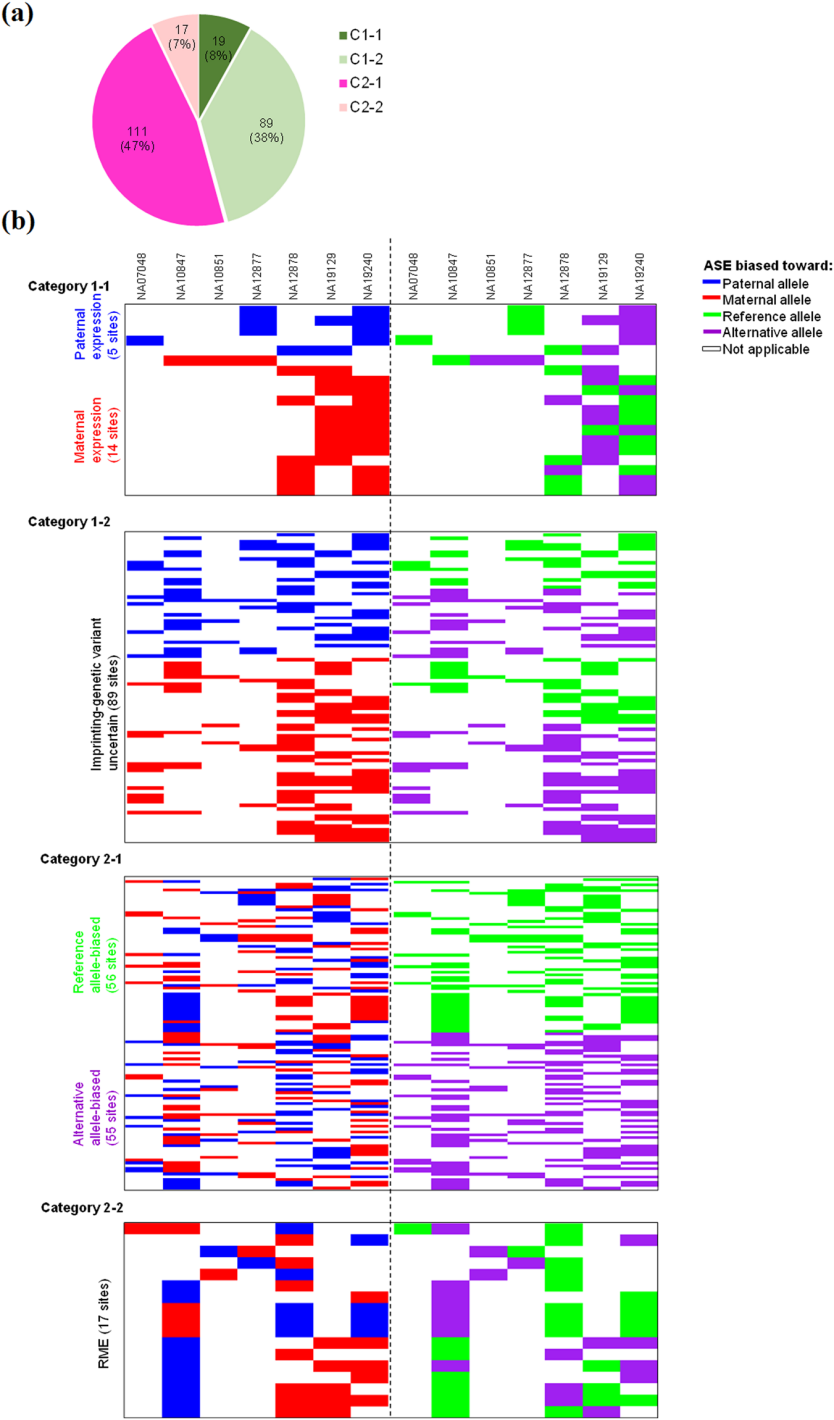
The four categories of ASE sites. (**a**) Distribution of the four categories. (**b**) The heatmap representing the distinct patterns for the four categories of ASE sites across the applicable individuals (see also Dataset 1 for further details).

To minimize varied confounding factors that result in ASE in specific individuals, all applicable individuals (at least two individuals) of the examined children should exhibit ASE at the heterozygous SNPs. After that, 236 ASE sites were extracted from the seven children. We dissected the distribution of these ASE sites among the applicable individuals and classified them into the following categories (Fig. 2a):

***Category 1*** **:** ***potential imprinting***

Category 1-1: Group 1 but neither Group 3 nor Group 4 ASE patterns were found across applicable individuals.

Category 1-2: Group 2 but no other Group (i.e., Groups 1, 3, and 4) ASE patterns were found across applicable individuals.

***Category 2*** **:** ***not imprinting***

Category 2-1:Group 3 but neither Group 1 nor Group 4 ASE patterns were found across applicable individuals.

Category 2-2:Group 4 ASE pattern was found across applicable individuals.

The heatmap represented the distinct patterns for these four categories of ASE sites across the applicable individuals (Fig. 2b). It was noteworthy that both Categories 1-1 and 2-1 ASE events exhibited the RAD pattern. ASE of Category 1-1 was very likely to be imprinting event as ASE consistently biased toward the same parental status without regard to genetic variant, whereas ASE of Category 2-1 was sequence-dependent without regard to allelic bias of parental status across heterozygous individuals. Obviously, the Category 2-1 ASE events were not derived from parent-of-origin allelic expression. On the other hand, although ASE of Category 1-2 also consistently biased toward the same allele across heterozygous individuals, we cannot determine which factor (imprinting or genetic variant) was the more likely explanation for the allelic expression bias. ASE of this category was thus considered imprinting-genetic variation uncertain. We found that >80% (89 out of 108) of Category 1 were imprinting-genetic variation uncertain (Fig. 2a), indicating the necessity of discriminating between imprinting- and genetic variation-dependent ASE events for Category 1-2. Regarding Category 2-2, since ASE did not bias toward any particular alleles across heterozygous individuals, RME may be a more likely explanation for ASE of this category. To minimize possible RME events in non-Categories 2-2, we excluded four ASE sites (two of Category 1-1: chromosome × 73823982 (*XIST*) and 130066326 (*ELF4*); two of Category 1-2: chromosome × 9716698 (*TBL1X*) and 73833838 (*XIST*)) located within the genes that contained other ASE sites of Category 2-2 and thus retained 232 ASE sites (17, 87, 111, and 17 sites for Categories 1-1, 1-2, 2-1, and 2-2, respectively; see also Dataset 1) for the following analyses.

### Evolutionary analysis of the four categories of ASE

**Figure 3:**
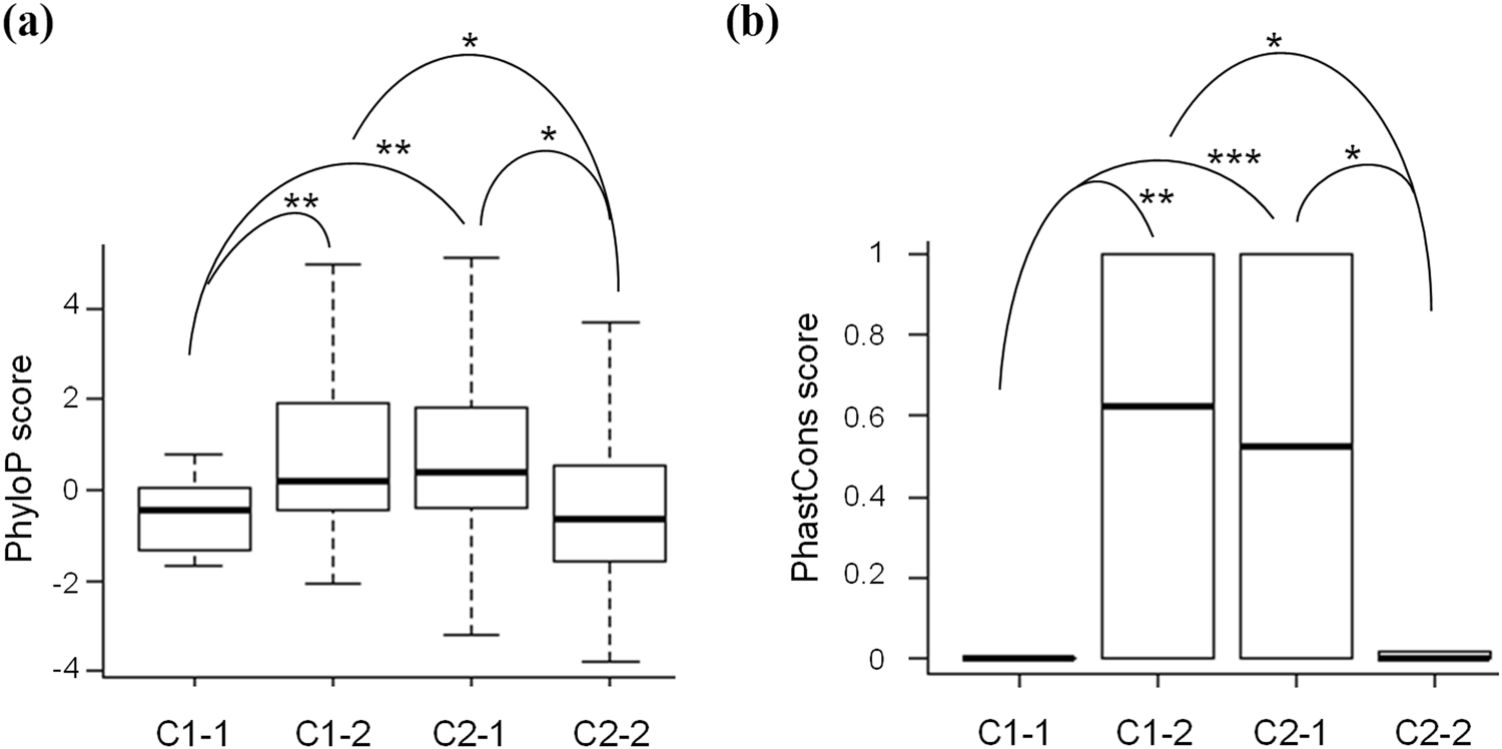
Comparisons of conservation scores measured by (**a**) PhyloP and (**b**) PhastCons for the four categories of ASE sites. The statistical significance was evaluated using the two-tailed Wilcoxon rank sum test (see also Supplemental Table S1). *P < 0.05, **P < 0.01, ***P < 0.001.

Previous studies have reported that purifying selection may act on expression variation^38–40^. Since monoallelic expression of Category 2-1 was highly sequence-dependent without regard to parent of origin, the sites of Category 2-1 were likely to be subject to functional constrains. In contrast, ASE of Categories 1-1 and 2-2 was independent of genetic variants; sites of these two categories should be subject to more relaxed selection pressure than those of Category 2-1. To this end, we examined how genetic variants may affect the conservation of sites (measured by the PhyloP^41^ and PhastCons^42^ scores) in these four categories. The sites of nonsynonymous variants (23 sites, ~10% of all examined sites) were excluded in this analysis, because these sites were expected to be under more stringent selective constraints than other sites. Figure 3 revealed that the PhyloP and PhastCons scores of the Category 2-1 sites were significantly higher than those of the Categories 1-1 and 2-2 sites (all *P* values < 0.05 by the two-tailed Wilcoxon rank sum test, see also Supplemental Table S1); and sites of Categories 1-1 and 2-2 had similar conservation scores (all *P* values > 0.05). As expected, this result suggested that the Category 2-1 sites were more conserved than the Categories 1-1 and 2-2 sites. Particularly note that, the conservation scores of the Category 1-2 sites, which were imprinting-genetic variation uncertain, were not statistically different from those of the Category 2-1 sites (all *P* values > 0.05) but significantly higher than those of the Categories 1-1 and 2-2 sites (all *P* values < 0.05, Fig. 3a and b). This result thus implied that, as compared with imprinting, genetic variation may be a more likely explanation for most ASE events of Category 1-2.

### Population-scale analysis of the four categories of ASE

**Figure 4:**
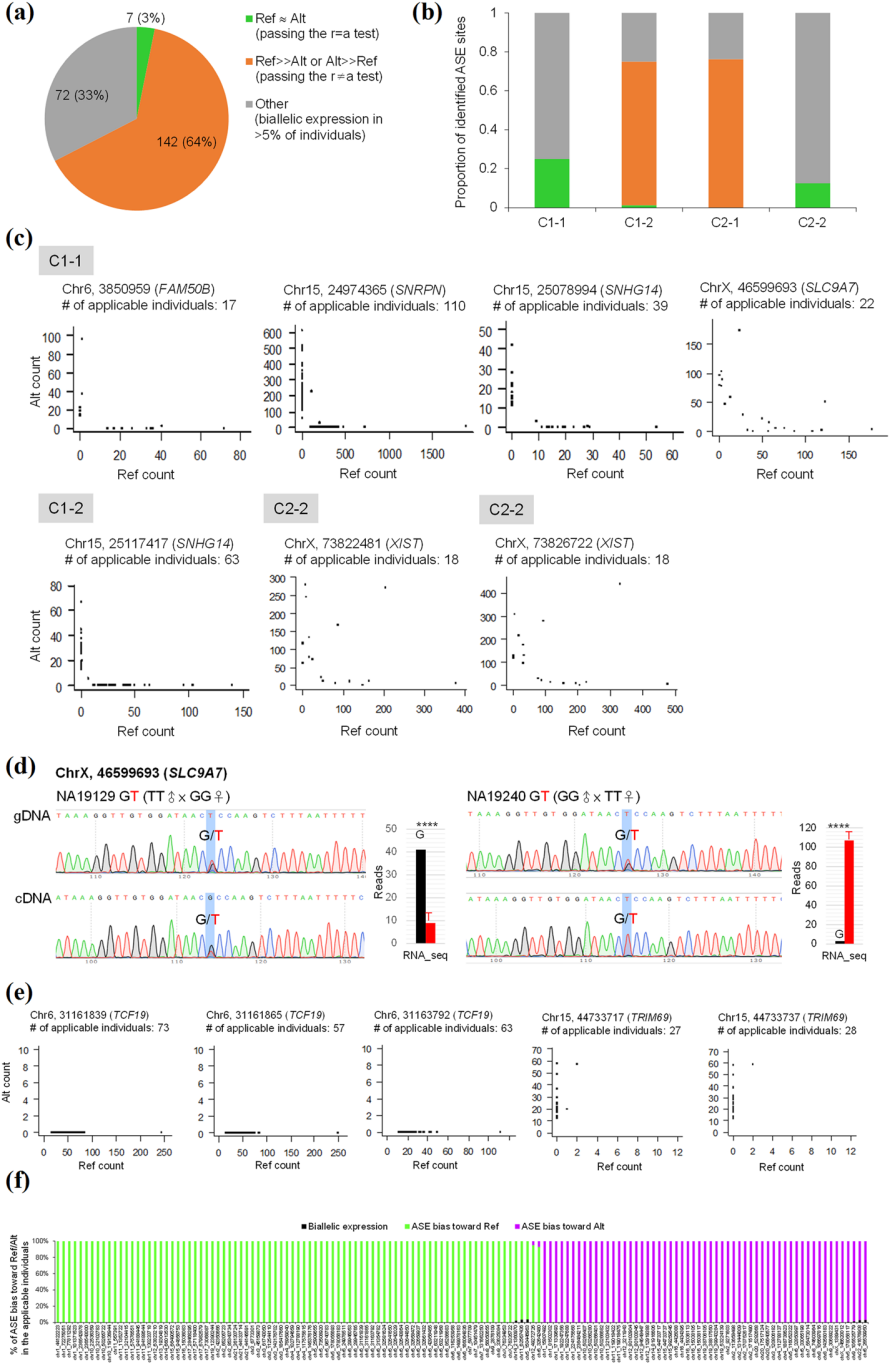
Population-scale analysis of the four categories of ASE in the Geuvadis LCL dataset. (**a** and **b**) Distribution of ASE variants passing the r = a test, ASE variants passing the r ≠ a test, and other ASE variants in (**a**) the total identified ASE variants and (**b**) the four categories of ASE. (**c**) Population-scale allelic expression patterns for the eight ASE variants passing the r = a test (five in Category 1-1, one in Category 1-2, and two in Category 2-2). Each plot depict represents the reference vs. alternative allele read counts for a SNP across all applicable individuals. A dot represents a SNP in an applicable individual. (**d**) Validation of the imprinting status of the newly identified imprinted gene (*SLC9A7*). Shown in the figure were the Sanger-sequencing results of *SLC9A7* with genomic DNA (gDNA) and cDNA from NA19129 and NA19240, which exhibited maternal expression with the RAD pattern between NA12878 and NA19240. Bases were colored as: A, green; C, blue; G, black; T, red. Significant differences between the paternally expressed read count and the maternally expressed read count (from the RNA-seq data) were evaluated using the *Chi*-square test. ****P < 0.0001. (**e**) Examples of population-scale allelic expression patterns for the ASE variants passing the r ≠ a test. (**f**) Distribution of ASE bias toward the reference allele, ASE bias toward the alternative allele, and biallelic expression in the applicable individuals for each ASE variant passing the r ≠ a test. A high-resolution figure (Fig. 4f) is provided in Dataset 1. Ref, the reference allele. Alt, the alternative allele.

Population-scale analyses have previously indicated that imprinted genes would exhibit ASE without a skew toward any particular alleles (i.e., reference or alternative alleles) among population^20^. These genes exhibit the transcriptional silencing of one allele and have monoallelic expression that evenly distributed between reference and alternative alleles across heterozygous individuals^20, 21^. In contrast, genetic variation-dependent ASE exhibits a consistent bias toward a particular allele (either the reference or the alternative alleles) in most heterozygous individuals^20^. Therefore, we measured ASE in the Geuvadis RNA-seq data from LCL in 261 individuals (134 females and 127 males)^34^ at the identified ASE sites of the four categories. Of the 232 ASE sites, we only considered the 221 sites, which were observed to be applicable SNPs (see Materials and Methods) in more than 10 Geuvadis LCL individuals. Of note, only the female individuals were considered if the ASE sites were located in chromosome X. To minimize false positives derived from partial imprinting or heterogeneous imprinting between individuals^19, 37^, the 149 ASE sites (67% of the 221 sites, Fig. 4a) that exhibited ASE in ≥95% of the applicable LCL individuals were extracted. We further divided the 149 ASE sites into two groups (see also Materials and Methods): (1) the ASE events passing “the r = a test” with monoallelic expression that nearly evenly distributed between the reference and alternative alleles across different individuals; and (2) the ASE events passing “the r ≠ a test” with monoallelic expression that had a significant bias toward either the reference or the alternative alleles across different individuals. The former can be regarded as imprinting events; whereas the latter were sequence-dependent and regarded to be genetic variation-dependent. Of the 149 ASE sites, only 7 sites passed the r = a test, whereas 142 sites passed the r ≠ a test (Fig. 4a and Supplemental Fig. S1), consistent with previous reports that genetic variation is a more likely cause of ASE than imprinting^43^. In terms of the four categories of ASE, we found that no events passing the r = a test were found in Category 2-1 and no events passing the r ≠ a test were found in Categories 1-1 and 2-2 (Fig. 4b and Supplemental Fig. S1). Of the 7 ASE sites passing the r = a test (Fig. 4c), the host genes of *FAM50B* ^14, 20, 21^, *SNRPN* ^21^, and *SNHG14* ^21^ were previously characterized to be maternally imprinted/paternally expressed in diverse human tissues. However, intriguingly, two Category 2-2 ASE sites located within *XIST* (X-inactive specific transcript), which is a well-known RME gene (i.e., random X-chromosome inactivation (XCI))^44, 45^, also passed the r = a test. This result revealed that RME genes may also exhibit monoallelic expression that evenly distributed between reference and alternative alleles across individuals, affecting the effectiveness of the population-based method for detecting imprinted genes. We thus suggest that our pipeline can help to distinguish RME events from imprinting ones if the Group 4 ASE pattern (Fig. 1c, right) is observed across different individuals. On the other hand, we found a Category 1-1 event passing the r = a test across Geuvadis LCL individuals (i.e., *SLC9A7*; Fig. 4c), which exhibited consistently maternal expression with the RAD pattern between NA19129 and NA19240 (Table 2). The existence of the heterozygosity and the monoallelic expression for this event in the corresponding LCL cell lines were experimentally validated by Sanger-sequencing with genomic DNA (gDNA) and cDNA of a single individual (Fig. 4d).

**Table 2:** The 7 ASE sites passing the r = a test.

Coordinate	Gene	Category	ASE pattern across the applicable children^*^							Note
			NA1	NA2	NA3	NA4	NA5	NA6	NA7	
Chr6, 3850959	*FAM50B*	C1-1	P_a_						P_r_	Paternal expression
Chr15, 24974365	*SNRPN*	C1-1				P_a_		P_r_	P_r_	Paternal expression
Chr15, 25078994	*SNHG14*	C1-1				P_a_			P_r_	Paternal expression
ChrX, 46599693	*SLC9A7*	C1-1						M_r_	M_a_	Maternal expression
Chr15, 25117417	*SNHG14*	C1-2		P_r_					P_r_	Paternal expression
ChrX, 73822481	*XIST*	C2-2		M_r_			P_a_		P_a_	RME
ChrX, 73826722	*XIST*	C2-2		M_r_			P_a_		P_a_	RME

*The ASE pattern (see also Fig. 1) of the children from the 7 trios illustrated in Fig. 1b: NA1, NA07048; NA2, NA10847; NA3, NA10851; NA4, NA12877; NA5, NA12878; NA6, NA19129; NA7, NA19240. A blank represents that the site of the corresponding individual is not applicable, e.g., heterozygous variant was not observed or the number of the mapped RNA-seq reads was not greater than ten on the site of the individual.

Particularly, the majority (76%) of ASE sites of Category 2-1 passed the r ≠ a test (Fig. 4b). The trend was also observed in ASE sites of Category 1-2, in which 74% of ASE sites passed the r ≠ a test (Fig. 4b). Only one site of Category 1-2 (which was located within a well-characterized imprinted gene, *SNHG14*) passed the r = a test (Fig. 4c). This result revealed that the population-scale allelic expression patterns of most Category 1-2 sites were similar to those of Category 2-1 sites, reflecting our abovementioned speculation that most ASE events of Category 1-2 were genetic variation-dependent. This also suggests that most ASE events without the RAD pattern, even though the allelic expression of these events are observed to consistently bias toward the same parental status across different family-based individuals, are not derived from imprinting. We thus suggest that the RAD pattern is a good indicator for discriminating between imprinting- and genetic variation-dependent ASE events.

Regarding the 142 ASE sites passing the r ≠ a test, the allelic expression remarkably biased toward a particular allele (either the reference or alternative alleles) in the overwhelming majority of individuals (see Fig. 4e and f and Supplemental Fig. S1). One may speculate that the monoallelic expression of these events is due to *cis*-regulation of QTL for ASE (the so-called “aseQTL”^40, 46^). However, if ASE of a heterozygous site is regulated by aseQTL, the heterozygous site will show biallelic expression when individuals exhibit homozygous at the aseQTL SNP^46^; by which some individuals will be biallelically expressed, while others will show ASE at the heterozygous site. Meanwhile, for the individuals who are heterozygous at the aseQTL SNP, an aseQTL-regulated site will not exhibit ASE with a skew toward a particular allele, which should exhibit even (or nearly even) distribution between the reference and alternative alleles across different individuals. Therefore, the *cis*-regulation of aseQTL seems not to be the explanation of the ASE sites passing the r ≠ a test. A possible explanation is that these ASE sites exhibit strong linkage disequilibrium with aseQTL SNPs^47^. Another possible explanation is that these ASE sites may themselves play a *cis*-regulatory role, because the ASE sites passing the r ≠ a test were more conserved than the sites failing to the r ≠ a test (Supplemental Fig. S2). The functional meaning of these events awaits further investigation.

### Potential caveats

**Figure 5:**
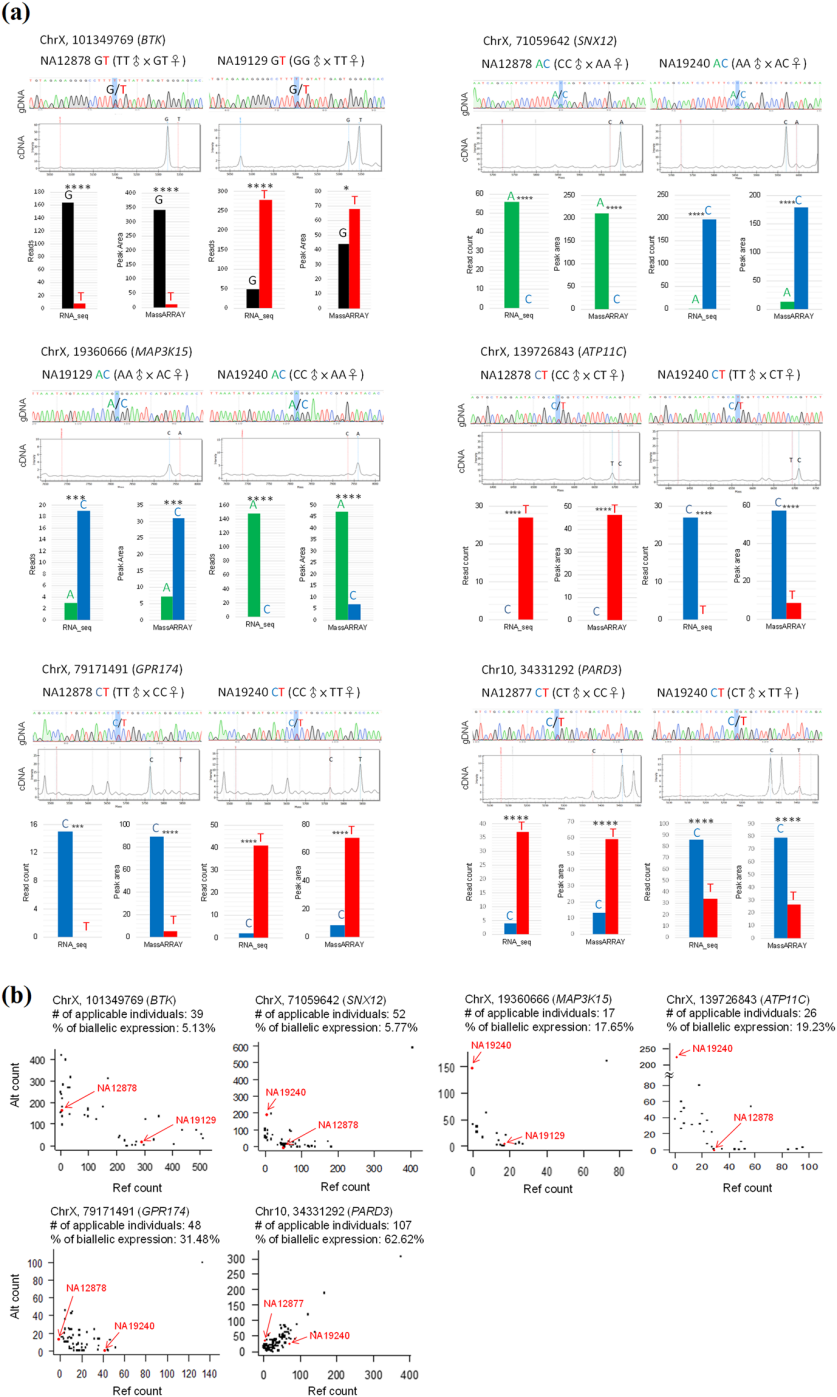
Validation of the Category 1-1 ASE sites failing to the r = a test. (**a**) Experimental validation of the existence of heterozygosity and the status of allelic expression for each site in the corresponding LCL cell lines using Sanger sequencing and MassARRAY platform. Bases were colored as: A, green; C, blue; G, black; T, red. Significant differences between the paternally expressed read count and the maternally expressed read count (from the RNA-seq data) and between the Peak areas (from the volume of peak in the MassARRAY data; see Dataset 3) were evaluated using the *Chi*-square test. ***P < 0.001, ****P < 0.0001. (**b**) Population-scale allelic expression patterns for the failing-test sites. “% of biallelic expression” represents the percentage of the Geuvadis individuals with biallelic expression at the failing-test sites. The red dots represent the allelic expression patterns of the failing-test sites between the corresponding heterozygous individuals listed in Fig. 1b (i.e., NA12877, NA12878, NA19129, or NA19240).

Of the four Category 1-1 sites that passed the r = s test, three were located within well-known imprinted genes and one was newly identified (Fig. 4c and Table 3). The monoallelic expression of the newly identified event (i.e., *SLC9A7*) has been confirmed (Fig. 4d). We are curious about why the failing-test Category 1-1 sites (13 sites) exhibited consistent expression biases toward the same parental status with the RAD pattern between the individuals listed in Fig. 1b but did not pass the r = a test in the population-scale analysis (Table 3 and Supplemental Fig. S1). We found that these sites failed to pass the r = s test because they showed biallelic expression in more than 5% of the heterozygous individuals of Geuvadis populations (Table 3). We questioned whether this inconsistence was due to the potential problems from analysis of the RNA-seq data (e.g., sequencing or alignment errors). To address this, we performed Sanger sequencing and MassARRAY analysis to confirm the existence of heterozygosity and the status of allelic expression for each Category 1-1 site in the corresponding LCL cell lines. As expected, all the four sites passing the r = s test were confirmed, each of which exhibited a consistent expression bias toward the same parentally inherited allele between the corresponding individuals in both the RNA-seq-based and MassARRAY results (Table 3 and Supplemental Fig. S3). This also provided another line of evidence supporting the newly identified imprinting event. In contrast, for the failing-test sites of Category 1-1, only 55% (6 out of 11 sites) were confirmed (Table 3, Fig. 5a, and Supplemental Fig. S3). Of note, the validation results of two sites (see Table 3) were not available because we failed to design appropriate primer sequences for the MassARRAY analysis. These results indicated that the Category 1-1 sites passing the r = s test were more accurate than the failing-test sites. Of the six Category 1-1 events, which did not pass the r = s test but were confirmed by the MassARRAY platform, one event (*PARD3*) exhibited remarkably paternal expression with the RAD pattern between NA12877 and NA19240; and the other (*BTK*, *SNX12*, *MAP3K15*, *ATP11C*, and *GPR174*) exhibited maternal expression with the RAD pattern between NA12878, NA19240, or NA19129 (Fig. 5a). Of note, NA12877, NA12878, and NA19240 are not involved in the Geuvadis populations. Regarding the population-scale allelic expression patterns for these ASE variants (see Fig. 5b; the red dots represented the allelic expression patterns of NA12877, NA12878, NA19129, or NA19240), two scenarios were observed. First, for *PARD3*, most (63%) of the heterozygous individuals of Geuvadis populations showed biallelic expression, suggesting that *PARD3* was a biallelically expressed gene. Second, in contrast, *BTK*, *SNX12*, *MAP3K15*, *ATP11C*, and *GPR174* showed biallelic expression in only the minority (5~31%) of the heterozygous individuals of Geuvadis populations but monoallelic expression with nearly even distribution between reference and alternative alleles in the other, suggesting that these genes were imprinting-dependent. Since these five genes are all located within chromosome X, we cannot eliminate the possibility that these events are subject to random XCI (see also Fig. 4c). A few clonal lines in the examined population showed monoallelic (or allele-biased) expression for biallelically expressed genes (the first scenario) or biallelic expression for imprinted/XCI genes (the second scenario) may be due to epigenetic instability in cloning, leading to aberrantly relaxed/repressive chromatin structure and then aberrantly allelic expression in specific individuals of clonal cell lines (particularly when the clonality level is high)^8, 20, 21, 48–51^.

**Table 3:** Validation of the Category 1-1 ASE sites using the population-based analysis (i.e., the r = s test) and the MassARRAY platform.

Coordinate		Gene	Known imprinted gene	Population-based analysis		Validated by MassARRAY
				% of biallelic expression^**a**^	Passing the r = s test	
chr6	3850959	*FAM50B*	Yes	0	Yes	Pass
chr15	24974365	*SNRPN*	Yes	0	Yes	Pass
chr15	25078994	*SNHG14*	Yes	2.56	Yes	Pass
chrX	46599693	*SLC9A7*	No	4.55	Yes	Pass
chrX	101349769	*BTK*	No	5.13	No	Pass
chrX	71059642	*SNX12*	No	5.77	No	Pass
chrX	12887911	*TLR7*	No	8.7	No	Fail
chrX	37811476	*CYBB*	No	13.16	No	Fail
chrX	19359763	*PDHA1*	No	15	No	Fail
chrX	19360666	*MAP3K15*	No	17.65	No	Pass
chrX	139726843	*ATP11C*	No	19.23	No	Pass
chrX	66597680	*EDA2R*	No	20	No	Fail
chrX	40606564	*ATP6AP2*	No	25	No	Fail
chr1	182383486	*GLUL*	No	26.74	No	NA^**b**^
chrX	79170974	*GPR174*	No	29.17	No	NA^**b**^
chrX	79171491	*GPR174*	No	31.48	No	Pass
chr10	34331292	*PARD3*	No	62.62	No	Pass

^a^The percentage of the Geuvadis individuals with biallelic expression at the variant sites.

^b^The validation results were not available because we failed to design appropriate primer sequences for the MassARRAY analysis.

## Discussion

We note that the RAD-based method is a compromise with the reciprocal mating approach. We have demonstrated that the RAD-based method is successfully applied to human, the species that a mating system of engineered crosses cannot be applied to. Like the reciprocal mating approach, our method detects allelic expression bias of parental status across independent parent-offspring trios, which requires the genotyping data from all members of the examined trio family and the corresponding RNA-seq data from the offspring of each trio family. The imprinting-dependent ASE events are then identified on the basis of only the SNP sites with the RAD pattern across at least two trio families (see Fig. 1a as an example). Therefore, the number of detected imprinting events is sensitive to the number of applicable SNPs across the examined heterozygous individuals. As accumulating family-based data is available in diverse tissues for species, the RAD-based method will be capable of detecting more novel imprinted genes, particularly tissue-dependent imprinting events, in the future.

Although the population-based method, which identifies imprinted genes by examining the distribution of ASE between the reference and alternative alleles across individuals, was reported to be effective for eliminating varied confounding factors that result in ASE in specific individuals^21, 22^, our results show that the RME effect may affect the effectiveness of the method for detecting imprinting events. We suggest that the Group 4 ASE pattern of our pipeline can help to distinguish between these two types of ASE events (see Fig. 4c for examples). In addition, population-based ASE analyses are often hampered by the paucity of both population-scale RNA-seq and genome-sequencing (or genotyping) data of a single individual.

As the clonality of the examined samples and the limited applicable SNPs with the RAD pattern between trio families, the aim of this study does not to identify many imprinting events in humans. We emphasize that the RAD pattern is a good indicator for categorization of varied types of ASE events. Particularly for the Category 1-2 ASE events, which exhibit a consistent expression bias toward the same parentally inherited allele between individuals (thereby they are often considered as imprinting events) but have no the RAD pattern, only 1% are indeed imprinting-dependent but more than 70% are genetic variation-dependent (Fig. 4b). We thus suggest that genetic variation is a more likely cause of ASE for Category 1-2 sites. While the functions of imprinting and RME events have long been established, the biological meanings of genetic variation-dependent ASE sites are relatively unclear. Imprinting events are known to be important in development and placental biology before birth^52^. RME events serve functions in dosage balance of X-linked genes between male and female cells or in immune cells and neurons^8^. For genetic variation-dependent ASE events (e.g., Categories 1-2 and 2-1 sites), we first show that they are more evolutionarily conserved than the other categories of sites (Fig. 3). We further find that these Categories 1-2 and 2-1 sites have a higher proportion of sites that share a linkage disequilibrium block with eQTL/aseQTL SNPs than the Categories 1-1 and 2-2 sites (Supplemental Fig. S4a and Dataset 2), suggesting that genetic variation-dependent ASE is actually linked with known eQTL/aseQTL events. Moreover, the Categories 1-2 and 2-1 sites tend to be in close proximity of CpG islands and gene regulatory elements such as Pol II and CTCF binding sequences (Supplemental Fig. S4b and Dataset 2). These observations thus support the role of genetic variation-dependent ASE events in *cis*-regulation.

In this study, we propose the RAD-based method, which is a compromise with the reciprocal mating approach and effective for discrimination between different types of ASE. We suggest that the RAD pattern should be taken into consideration while detecting the status of parentally inherited imprinting, even though family data sets are used. Our findings thus help to increase our understanding of monoallelic expression, expanding this widespread and complex mechanism for comprehensive characterization in the transcriptomes.

## Materials and Methods

### Data retrieval and availability

The SNP information and genotyping data of the seven trio families used (Fig. 1b) were downloaded from the HapMap project at ftp://ftp.ncbi.nlm.nih.gov/hapmap/hapmart/2009-05_rel27/, which were also collected in the 1000 genome project at http://browser.1000genomes.org/. The corresponding RNA-seq data of the seven children came from three studies: (1) the Geuvadis RNA Sequencing Project at http://www.geuvadis.org/web/geuvadis/rnaseq-project (for NA07048, NA10847, NA10851, and NA19129; accession number: ERP001942)^34^; (2) the Li *et al*.’s study (for NA12877 and NA12878; accession number: GSM1372330 and GSM1372331)^35^; and (3) the Cenik *et al*.’s study (for NA19240; accession number: GSE65912)^36^. The human genomic sequences (GRCh38) and annotation were downloaded from the GENCODE project (Release 24) at http://www.gencodegenes.org/. The Geuvadis RNA-seq data from LCL in 261 individuals (134 females and 127 males)^34^ were downloaded from the Geuvadis RNA Sequencing Project. The corresponding genotyping data of the LCL samples were also downloaded from the HapMap project. The 261 samples were selected as the genotyping data of these samples were stored in both the HapMap and 1000 genome projects. The *cis*-eQTLs identified from HapMap human LCLs were download from the seeQTL database^53^. The aseQTLs were retrieved from Battle *et al*.s’ stdudy^46^. The linkage disequilibrium based haplotype blocks (linkage disequilibrium blocks) were calculated by the R package LDExplorer^54^ on the basis of the three human populations (African, European, and Asian) from the 1000 Genome project and downloaded from the LDExplorer website at http://www.eurac.edu/en/research/health/biomed/services/Pages/LDExplorer.aspx. The CpG islands were downloaded from the UCSC genome browser at https://genome.ucsc.edu/. The regulatory elements such as Pol II/CTCF/transcription factor binding sequences and enhancer elements were retrieved from the Ensembl genome browser^55^ at http://www.ensembl.org/ (release 84). The identified Categories 1-1, 1-2, 2-1, and 2-2 ASE sites and related information are illustrated in Datasets 1 and 2. The Sanger sequencing primers, MassARRAY primers, and MassARRAY results are listed in Dataset 3.

### Identification of ASE sites

To extract SNPs of ASE with the RAD pattern (Fig. 1a), we first retrieved seven family trios (Fig. 1b), in which all members of each trio family were genotyped and the corresponding RNA-seq data of the child of each trio family should be available. We then masked the SNP sites and generated the pseudo genome for each child using the maskOutFa tool (https://github.com/ENCODE-DCC/kentUtils/tree/master/src/hg/maskOutFa). The corresponding RNA-seq reads were then aligned against the generated pseudo genome for each child using STAR (version 2.5.2a)^56^. SAMtools mpileup^57^ and the perl procedure of pileup2base (https://github.com/riverlee/pileup2base/blob/master/pileup2base.pl) were used to call variant bases at the specified SNP sites. For each child, an applicable SNP (Table 1) was considered informative for analysis only if it satisfied all the following criteria: (1) the site should be a heterozygous SNP located within a genic region; (2) at least one of his/her parents was homozygous; (3) the type of dimorphic nucleotide pattern for this SNP site should be the same in all heterozygous individuals examined; (4) the number of the mapped RNA-seq reads should be greater than 10; and (5) if more than two nucleotide types were called, the third allele should be less than 10% of the mapped reads. The ASE sites were determined by using the *Chi*-square test (*P* values < 0.05) on the mapped reads at the applicable SNPs. For the population-scale analysis, the corresponding RNA-seq reads were first aligned against the maskOutFa-generated pseudo genome for each Geuvadis individual using STAR. The ASE status of the identified 236 ASE sites across the 261 Geuvadis individuals was then determined by the similar manner stated above. Twelve ASE sites were not considered as they were not applicant in more than 10 LCL heterozygous individuals. The population-scale allelic expression patterns for the 224 sites were provided in Supplemental Fig. S1. To minimize the false positives derived from partial imprinting or heterogeneous imprinting between individuals^19, 37^, we focused on the 150 ASE sites (Fig. 4a) that exhibited ASE in ≥95% of the applicable LCL individuals. Of note, only the female individuals were considered if the ASE sites were located in chromosome X. An ASE event was considered to be imprinting- or RME-dependent, if it satisfied that the ratio of the number of the applicable individuals with an expression bias toward the reference allele to that with an expression bias toward the alternative allele was not statistically different from one (i.e., the r = a test, with *P* value > 0.05 by the *Chi*-square test); otherwise, it was regarded to be genetic variation-dependent (i.e., the r ≠ a test, with *P* value < 0.05).

### Cell culture and validation of allelic expression

The commercial LCL cell lines of the seven children (NA07048, NA10847, NA10851, NA12877, NA12878, NA19129 and NA19240) were obtained from the Coriell Institute for Medical Research. All cell lines were cultured in RPMI1640 medium (Thermo Fisher Scientific) supplemented with 10% FBS and Antibiotic-Antimycotic (Thermo Fisher Scientific). The pallets were stocked on −20 °C. All cells were tested to be free of Mycoplasma using EZ-PCR Mycoplasma Test Kit (Biological Industries). The PureLink Genomic DNA Mini Kit (Thermo Fisher Scientific) and PureLink RNA Mini Kit (Thermo Fisher Scientific) were used to isolate genomic DNA and RNA, respectively. cDNA was prepared from 5 μg total RNA with SuperScript III First-Strand Synthesis System (Thermo Fisher Scientific) using Random Hexamer and Oligo-dT primers. PCR was performed using DreamTaq Green PCR Master Mix (Thermo Fisher Scientific) on Veriti Thermal Cycler (Thermo Fisher Scientific). PCR products were validated by gel and then treated with QIAquick Gel Extraction Kit (Qiagen). All PCR products were performed on the Sanger sequencing platform using 3730xl DNA Analyzers (Thermo Fisher Scientific). For MassARRAY analysis, allelic expression of the Sanger sequencing-confirmed variants was validated by Sequenom MassARRAY assay. The SNP typing was performed by Agena MassARRAY with iPLEX pro chemistry (Agena Bioscience). By following the manufacture guide^58^, the Assay Designer software package (v.4.0) was used to design the specific PCR primer and MassEXTEND primer sequences. The cDNA sample (1 µl) was then applied to mutiplex PCR reaction in 5 µl volumes containing 1 unit of Taq polymerase, 500 nmol of each PCR primer mix, and 2.5 mM of each dNTP (Agena, PCR accessory and Enzyme kit). The Sequenom MALDI-TOP iPLEX experiments and analyses were performed by FENG CHI Biotech Corporation. Purified primer extension reaction was added into a matrix pad of a SpectroCHIP (Agena Bioscience). SpectroCHIPs detection and the calling by clustering analysis were performed using MassARRAY Analyzer 4 and TYPER 4.0 software, respectively. Significant differences between the Peak areas (the volume of peak) were evaluated using the *Chi*-square test.

## Electronic supplementary material

Supplementary Information

Dataset 1

Dataset 2

Dataset 3

## References

[1] Lim DH, Maher ER (2010). Genomic imprinting syndromes and cancer. Adv. Genet..

[2] Ishida M, Moore GE (2013). The role of imprinted genes in humans. Mol. Aspects Med..

[3] Perez JD, Rubinstein ND, Dulac C (2016). New Perspectives on Genomic Imprinting, an Essential and Multifaceted Mode of Epigenetic Control in the Developing and Adult Brain. Annu. Rev. Neurosci..

[4] Babak T (2012). Identification of imprinted loci by transcriptome sequencing. Methods Mol. Biol..

[5] Babak T (2008). Global survey of genomic imprinting by transcriptome sequencing. Curr. Biol..

[6] Wang X (2008). Transcriptome-wide identification of novel imprinted genes in neonatal mouse brain. PLoS One.

[7] Gregg C (2010). High-resolution analysis of parent-of-origin allelic expression in the mouse brain. Science.

[8] Reinius B, Sandberg R (2015). Random monoallelic expression of autosomal genes: stochastic transcription and allele-level regulation. Nat. Rev. Genet..

[9] Chess A (2012). Mechanisms and consequences of widespread random monoallelic expression. Nat. Rev. Genet..

[10] Nica AC, Dermitzakis ET (2013). Expression quantitative trait loci: present and future. Philos. Trans. R. Soc. Lond B. Biol. Sci..

[11] Goring HH (2007). Discovery of expression QTLs using large-scale transcriptional profiling in human lymphocytes. Nat. Genet..

[12] Stranger BE (2007). Relative impact of nucleotide and copy number variation on gene expression phenotypes. Science.

[13] Dixon AL (2007). A genome-wide association study of global gene expression. Nat. Genet..

[14] Luedi PP (2007). Computational and experimental identification of novel human imprinted genes. Genome Res..

[15] Kanber D (2009). The human retinoblastoma gene is imprinted. PLoS Genet..

[16] Monk D (2008). Comparative analysis of human chromosome 7q21 and mouse proximal chromosome 6 reveals a placental-specific imprinted gene, TFPI2/Tfpi2, which requires EHMT2 and EED for allelic-silencing. Genome Res..

[17] Yu Y (1999). NOEY2 (ARHI), an imprinted putative tumor suppressor gene in ovarian and breast carcinomas. Proc. Natl. Acad. Sci..

[18] Okita C (2003). A new imprinted cluster on the human chromosome 7q21-q31, identified by human-mouse monochromosomal hybrids. Genomics.

[19] Morcos L (2011). Genome-wide assessment of imprinted expression in human cells. Genome Biol..

[20] Babak T (2015). Genetic conflict reflected in tissue-specific maps of genomic imprinting in human and mouse. Nat. Genet..

[21] Baran Y (2015). The landscape of genomic imprinting across diverse adult human tissues. Genome Res..

[22] Castel SE, Levy-Moonshine A, Mohammadi P, Banks E, Lappalainen T (2015). Tools and best practices for data processing in allelic expression analysis. Genome Biol..

[23] Crowley JJ (2015). Analyses of allele-specific gene expression in highly divergent mouse crosses identifies pervasive allelic imbalance. Nat. Genet..

[24] Pinter SF (2015). Allelic Imbalance Is a Prevalent and Tissue-Specific Feature of the Mouse Transcriptome. Genetics.

[25] Leung D (2015). Integrative analysis of haplotype-resolved epigenomes across human tissues. Nature.

[26] Roadmap Epigenomics C (2015). Integrative analysis of 111 reference human epigenomes. Nature.

[27] Heinz S (2013). Effect of natural genetic variation on enhancer selection and function. Nature.

[28] Kasowski M (2013). Extensive variation in chromatin states across humans. Science.

[29] Kilpinen H (2013). Coordinated effects of sequence variation on DNA binding, chromatin structure, and transcription. Science.

[30] DeVeale B, van der Kooy D, Babak T (2012). Critical evaluation of imprinted gene expression by RNA-Seq: a new perspective. PLoS Genet..

[31] Chess A (2016). Monoallelic Gene Expression in Mammals. Annu. Rev. Genet..

[32] Genomes Project C (2015). A global reference for human genetic variation. Nature.

[33] Sudmant PH (2015). An integrated map of structural variation in 2,504 human genomes. Nature.

[34] Lappalainen T (2013). Transcriptome and genome sequencing uncovers functional variation in humans. Nature.

[35] Li X (2014). Transcriptome sequencing of a large human family identifies the impact of rare noncoding variants. Am. J. Hum. Genet..

[36] Cenik C (2015). Integrative analysis of RNA, translation, and protein levels reveals distinct regulatory variation across humans. Genome Res..

[37] Wolf JB, Cheverud JM, Roseman C, Hager R (2008). Genome-wide analysis reveals a complex pattern of genomic imprinting in mice. PLoS Genet..

[38] Ronald J, Akey JM (2007). The evolution of gene expression QTL in Saccharomyces cerevisiae. PLoS One.

[39] Rockman MV, Skrovanek SS, Kruglyak L (2010). Selection at linked sites shapes heritable phenotypic variation in C. elegans. Science.

[40] Josephs EB, Lee YW, Stinchcombe JR, Wright SI (2015). Association mapping reveals the role of purifying selection in the maintenance of genomic variation in gene expression. Proc. Natl. Acad. Sci..

[41] Pertea M, Pertea GM, Salzberg SL (2011). Detection of lineage-specific evolutionary changes among primate species. BMC Bioinformatics.

[42] Siepel A (2005). Evolutionarily conserved elements in vertebrate, insect, worm, and yeast genomes. Genome Res..

[43] Zhang K (2009). Digital RNA allelotyping reveals tissue-specific and allele-specific gene expression in human. Nat. Methods.

[44] Brown CJ (1991). A gene from the region of the human X inactivation centre is expressed exclusively from the inactive X chromosome. Nature.

[45] Brockdorff N (1991). Conservation of position and exclusive expression of mouse Xist from the inactive X chromosome. Nature.

[46] Battle A (2014). Characterizing the genetic basis of transcriptome diversity through RNA-sequencing of 922 individuals. Genome Res..

[47] Raghavan, A. *et al*. High-throughput Screening and CRISPR-Cas9 Modeling of Causal Lipid-associated Expression Quantitative Trait Locus Variants. *bioRxiv* (2016).

[48] Haigh AJ, Lloyd VK (2006). Loss of genomic imprinting in Drosophila clones. Genome.

[49] Mekhoubad S (2012). Erosion of dosage compensation impacts human iPSC disease modeling. Cell Stem Cell.

[50] Nazor KL (2012). Recurrent variations in DNA methylation in human pluripotent stem cells and their differentiated derivatives. Cell Stem Cell.

[51] Stadtfeld, M. *et al*.. Ascorbic acid prevents loss of Dlk1-Dio3 imprinting and facilitates generation of all-iPS cell mice from terminally differentiated B cells. *Nat. Genet*. **44**, 398–405 S391–392 (2012). 10.1038/ng.1110 PMC3538378 22387999

[52] Peters J (2014). The role of genomic imprinting in biology and disease: an expanding view. Nat. Rev. Genet..

[53] Xia K (2012). seeQTL: a searchable database for human eQTLs. Bioinformatics.

[54] Taliun D, Gamper J, Pattaro C (2014). Efficient haplotype block recognition of very long and dense genetic sequences. BMC Bioinformatics.

[55] Zerbino DR, Wilder SP, Johnson N, Juettemann T, Flicek PR (2015). The ensembl regulatory build. Genome Biol..

[56] Dobin A (2013). STAR: ultrafast universal RNA-seq aligner. Bioinformatics.

[57] Li H (2009). The Sequence Alignment/Map format and SAMtools. Bioinformatics.

[58] Gabriel S, Ziaugra L, Tabbaa D (2009). SNP genotyping using the Sequenom MassARRAY iPLEX platform. Curr. Protoc. Hum. Genet..

