# Supplementary material for: Assessment of imprinting- and genetic variation-dependent monoallelic expression using reciprocal allele descendants between human family trios
Source: Sci Rep. 2017 Aug 1;7:7038. doi: 10.1038/s41598-017-07514-z (PMC5539102; doi:10.1038/s41598-017-07514-z)
Supplement: Supplementary file 1 — Supplementary Information [file 41598_2017_7514_MOESM1_ESM.doc]

**Assessment of imprinting- and genetic variation-dependent monoallelic expression using reciprocal allele descendants between human family trios**

**Trees-Juen Chuang1[[1]](#footnote-2)*, Yu-Hsiang Tseng ^1^, Chia-Ying Chen^1^, Yi-Da Wang^1^**

^1^Genomics Research Center, Academia Sinica, Taipei, Taiwan

**Supplementary Information**

**The supplementary file includes one supplemental table (Table S1) and four supplemental figures (Figs. S1-S4).**

**Supplemental Table S1.** Significant test on PhyloP (red words; upper right) and PhastCons (black words; lower left) scores for the differences between the Categories 1-1, 1-2, 2-1, and 2-2 ASE sites by using the two-tailed Wilcoxon rank sum test, related to Figure 3. NS, not significant.

**(a) Category 1-1**

- Passing the r=a test: 4 sites


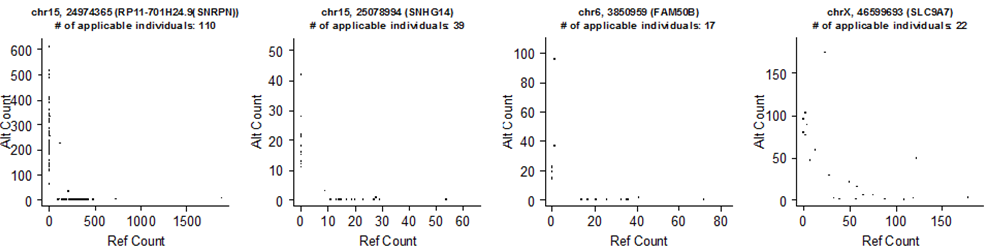


- Passing the ra test: 0 site
- Other: 12 sites


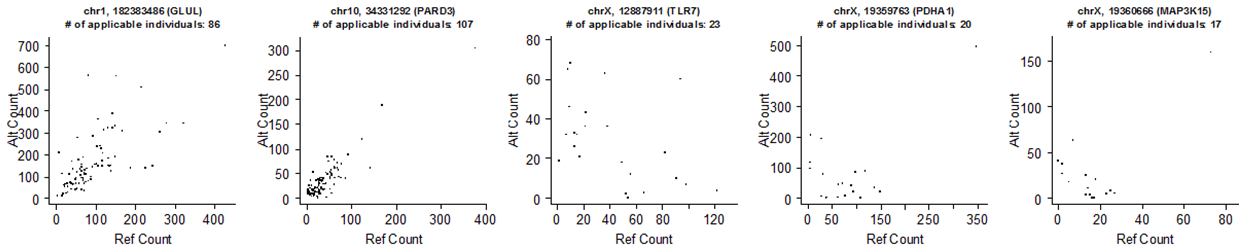


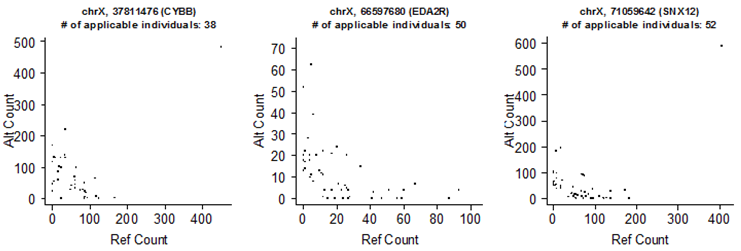

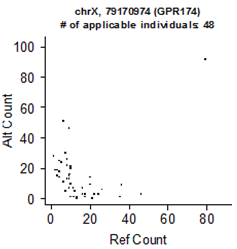

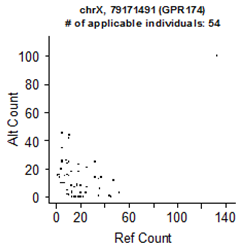


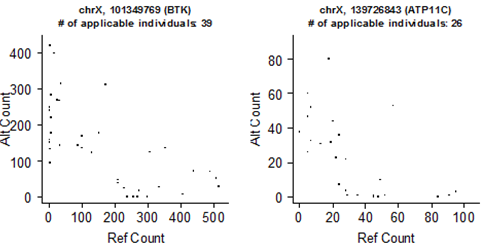


**(b) Category 1-2**

- Passing the r=a test: 1 site


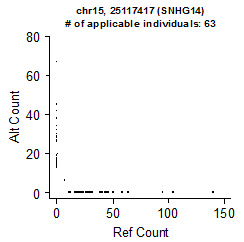


- Passing the ra test: 62 sites


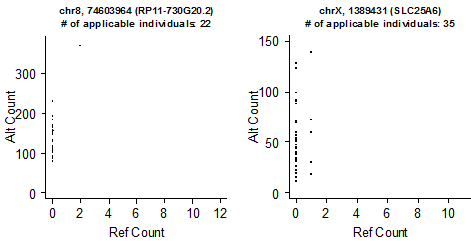


- Other: 21 sites


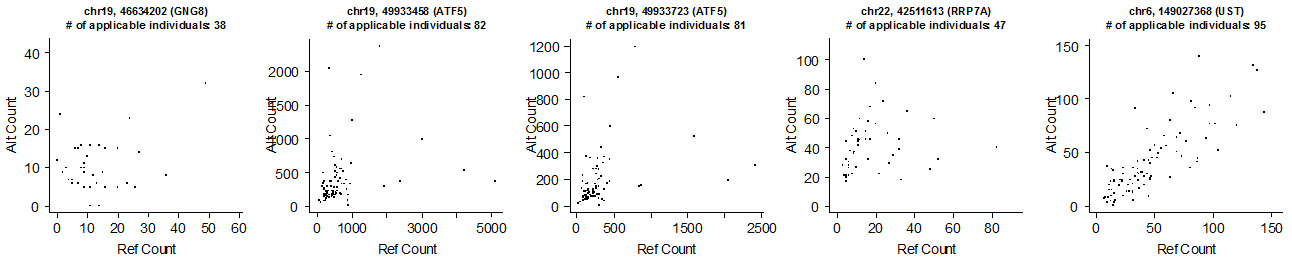

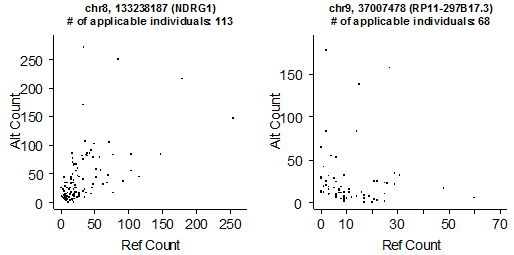


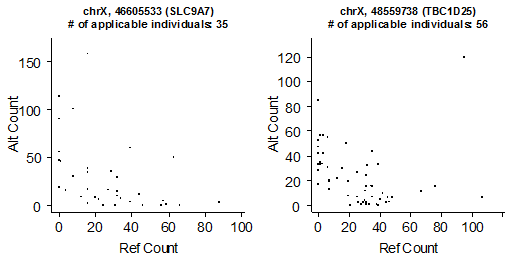

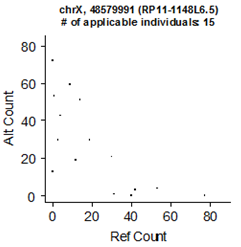


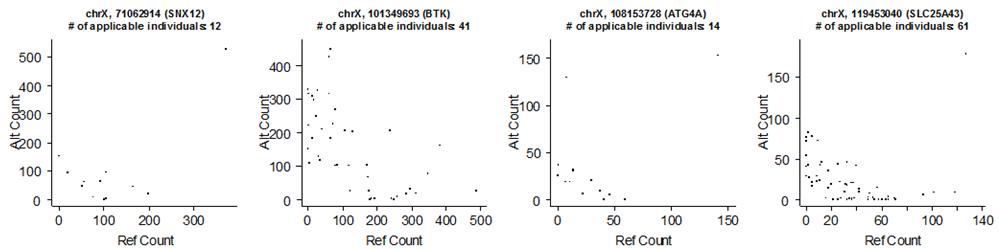

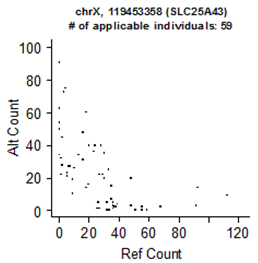


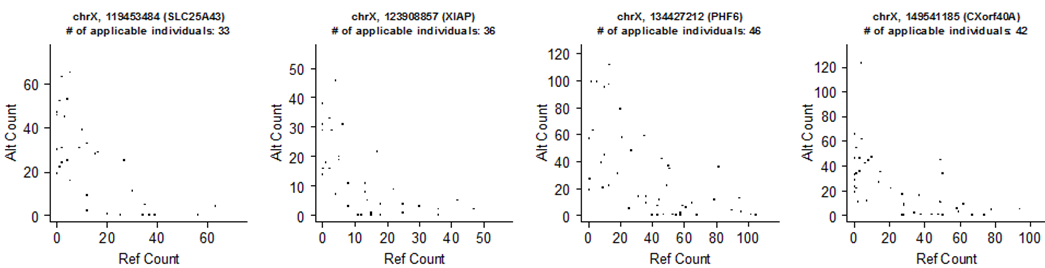

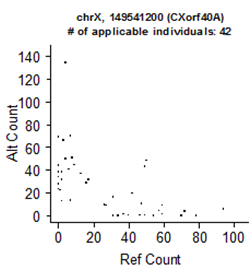


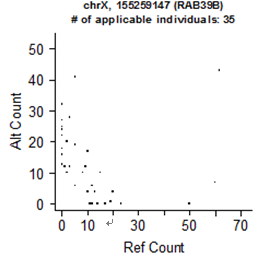


**(c) Category 2-1**

- Passing the r=a test: 0 site
- Passing the ra test: 80 sites


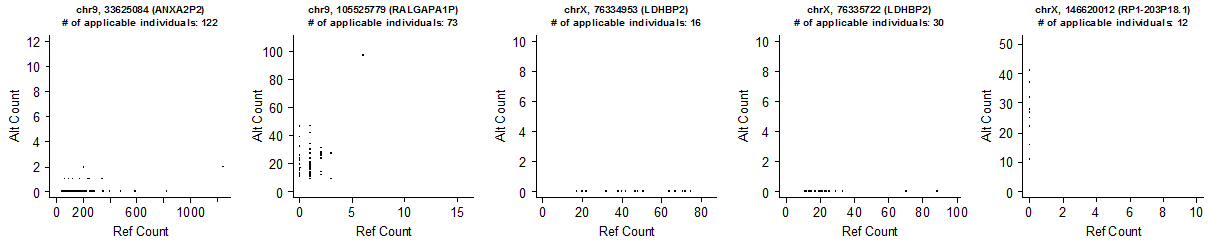


- Other: 25 sites

**
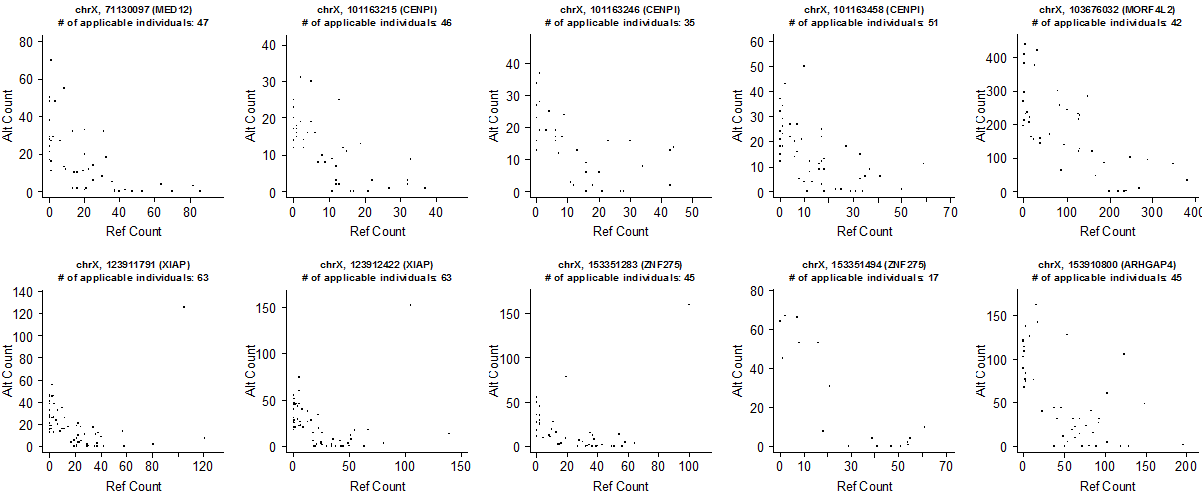
**

**(d) Category 2-2**

- Passing the r=a test: 2 sites


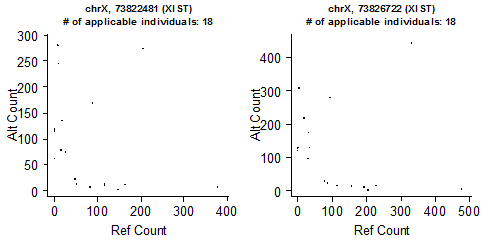


- Passing the ra test: 0 site
- Other: 14 sites

**Supplemental Figure S1**. Population-scale allelic expression patterns for ASE variants passing the r=a test, ASE variants passing the ra test, and other ASE variants in (a) Category 1-1, (b) Category 1-2, (c) Category 2-1, and (d) Category 2-2. Each plot depict represents the reference vs. alternative allele read counts for a SNP across all applicable individuals. A dot represents a SNP in an applicable individual.

**(a) (b)**

**Supplemental Figure S2.** Comparisons of (a) PhyloP and (b) PhastCons scores of ASE sites passing the r=a test, ASE sites passing the ra test, and other ASE sites. The statistical significance was evaluated using the two-tailed Wilcoxon rank sum test. **P*<0.05, *****P*<0.0001, NS, not significant.

**Supplemental Figure S3.** Validation results of the existence of heterozygosity and the status of allelic expression for each Category 1-1 sites in the corresponding LCL cell lines using Sanger sequencing and MassARRAY platform. Bases were colored as: A, green; C, blue; G, black; T, red. Significant differences between the paternally expressed read count and the maternally expressed read count (from the RNA-seq data) and between the Peak areas (from the volume of peak in the MassARRAY data) were evaluated using the *Chi*-square test. **P*<0.05, ***P*<0.01, ****P*<0.001, *****P*<0.0001.

**(a)**

**(b)**

**Supplemental Figure S4.** Comparisons of the percentages of sites (a) that share a linkage disequilibrium (LD) block (in African, European, and Asian populations) with eQTL/aseQTL SNPs and (b) that are in close proximity of CpG islands and gene regulatory elements (≤ 2k bp) such as Pol II/CTCF/transcription factor (TF) binding sequences and enhancer elements for the four categories of ASE sites. Our results reveal that Categories 1-2 and 2-1 sites tend to be in close proximity of CpG islands and Pol II and CTCF binding sequences.

1. ^*^ To whom correspondence should be addressed. E-mail: trees@gate.sinica.edu.tw (TJC). [↑](#footnote-ref-2)
